# A Comparative Proteomic Analysis of *Pinellia ternata* Leaves Exposed to Heat Stress

**DOI:** 10.3390/ijms141020614

**Published:** 2013-10-15

**Authors:** Yunhao Zhu, Guosheng Zhu, Qiaosheng Guo, Zaibiao Zhu, Changlin Wang, Zuoyi Liu

**Affiliations:** 1 Institute of Chinese Medicinal Materials, Nanjing Agricultural University, Nanjing 210095, China; E-Mails: guxinhan123@163.com (Y.Z.); zhuzaibiao@njau.edu.cn (Z.Z.); wangcl@njau.edu.cn (C.W.); 2 Institute of Morden Chinese Medical Materials, Guizhou Academy of Agricaltural Sciences, Guiyang 550006, China; E-Mail: zgsah@163.com; 3 Key Laboratory of Agricultural Biotechnology, Guizhou Academy of Agricultural Sciences, Guiyang 550006, China

**Keywords:** proteomics, quantitative real-time PCR, heat stress, *Pinellia ternata*

## Abstract

*Pinellia ternata* is an important traditional Chinese medicinal plant. The growth of *P. ternata* is sensitive to high temperatures. To gain a better understanding of heat stress responses in *P. ternata*, we performed a comparative proteomic analysis. *P. ternata* seedlings were subjected to a temperature of 38 °C and samples were collected 24 h after treatment. Increased relative ion leakage and lipid peroxidation suggested that oxidative stress was frequently generated in rice leaves exposed to high temperature. Two-dimensional electrophoresis (2-DE) was used to analyze heat-responsive proteins. More than 600 protein spots were reproducibly detected on each gel; of these spots, 20 were up-regulated, and 7 were down-regulated. A total of 24 proteins and protein species were successfully identified by MALDI-TOF/TOF MS. These proteins and protein species were found to be primarily small heat shock proteins (58%) as well as proteins involved in RNA processing (17%), photosynthesis (13%), chlorophyll biosynthetic processes (4%), protein degradation (4%) and defense (4%). Using 2-DE Western blot analysis, we confirmed the identities of the cytosolic class II small heat shock protein (sHSPs-CII) identified by MS. The expression levels of four different proteins [cytosolic class I small heat shock protein (sHSPs-CI), sHSPs-CII, mitochondrial small heat shock protein (sHSPs-MIT), glycine-rich RNA-binding protein (GRP)] were analyzed at the transcriptional level by quantitative real-time PCR. The mRNA levels of three sHSPs correlated with the corresponding protein levels. However, GRP was down-regulated at the beginning of heat stress but then increased substantially to reach a peak after 24 h of heat stress. Our study provides valuable new insight into the responses of *P. ternata* to heat stress.

## 1. Introduction

Temperature stress is becoming more severe due to global warming, and high temperature has become a major deleterious factor affecting plant productivity. Heat stress disturbs cellular homeostasis and can lead to severe retardation in growth and development and even death [1]. Temperature stress can also have a devastating effect on plant metabolism, and thus, studying how plants respond to high temperature stress is important to facilitate the development of heat-tolerant cultivars.

One heat-stress response that is ubiquitous among plants is the expression of heat shock proteins (HSPs), and this response is known to be an important adaptive strategy in plant tolerance to heat stress [2]. These proteins in plants are grouped into five classes according to their approximate molecular weight: (1) Hsp100, (2) Hsp90, (3) Hsp70, (4) Hsp60 and (5) small heat shock proteins (sHSPs) [3,4]. sHSPs are the most abundant heat stress-induced proteins in higher plants, in which they are molecular chaperones that play a protective role against various stresses. In plants, a large number of HSPs that are expressed in response to heat stress have been identified; however, considerable variation exists in the HSPs produced among different plant species and even among individuals of the same species [5].

Proteomics is a powerful molecular tool that is used to compare proteomes under different conditions, such as heat stress or other stressful conditions. Proteomic responses to heat stress have been investigated in a number of plants, such as rice (*Oryza sativa* L.) [6,7]; wheat (*Triticum aestivum* L.) [8–10]; barley (*Hordeum vulgare* L.) [11], and maize (*Zea mays*) [12,13], while many stress-related proteins have been identified. A heat-induced increase in several HSPs including proteins from HSP100, HSP70 and sHSP families has been observed. sHSPs belonging to cytoplasmic-located sHSPs as well as mitochondrial-targeted and chloroplast-targeted sHSPs were detected. Another characteristic feature of heat stress is oxidative damage [14]. Up-regulation of several enzymes involved in redox homeostasis such as GST, chloroplast precursors of SOD was reported [15]. In addition, increased accumulation of some eukaryotic translation initiation factors indicates profound cellular reorganisation leading to programmed cell death (PCD) under long-term heat treatment. However, most heat-related proteomic analyses have concentrated on food crops. The thermo-tolerance of medicinal plants at the proteomic level has not been well investigated, and the molecular basis is poorly understood.

*P. ternata* is a perennial medicinal herb that grows primarily in China. It is an important traditional Chinese medicinal plant and has been used widely for thousands of years in China. Its tuber has specific pharmacological properties, such as antiemetic, antiobesity, expectorant, antipyretic and styptic effects, among others [16,17]. *P. ternata* has been excessively exploited, which has resulted in a continual decrease in wild sources of the species annually. The growth of *P. ternata* is sensitive to variations in temperature. Sprout tumble occurs gradually on a large-scale when the temperature rises to over 35 °C, leading to a reduction in its output in agricultural production [18]. Understanding the mechanisms of sprout tumble formation in *P. ternata* will provide important information concerning protective mechanisms against high temperature that may be present in other temperature-sensitive organisms.

In the present study, we analyzed the changes in the total proteins in *P. ternata* leaves after heat treatment. We found 27 differentially expressed spots and identified 24 proteins and protein species by two-dimensional electrophoresis (2-DE) in combination with MALDI-TOF/TOF. The accuracy of the 2-DE analysis was further validated by Western blot analysis, and real-time quantitative PCR (qPCR) was used to determine whether the differential expression levels of four selected proteins correlated with transcript abundance. This study offers insights into the physiological mechanisms of the response to high temperatures.

## 2. Results

### 2.1. Physiological Responses Induced by Heat Stress

**Figure 1:**
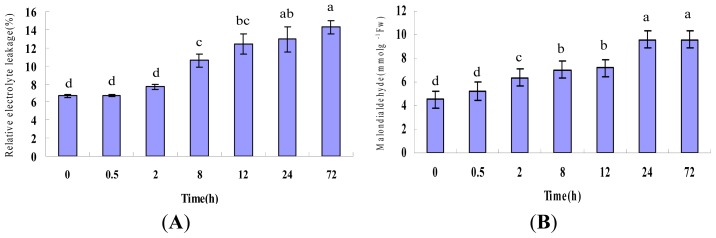
Physiological responses of *P. ternata* leaf under heat stress. (**A**) Relative electrolyte leakage; (**B**) Malondialdehyde (MDA) content of *P. ternata* leaves after heat treatments. Each point represents the average of three individual identical experiments (±SD). Letters above the bars (a, b, c, d, ab and bc) indicate a statistically significant difference (*p* < 0.05) according to Duncan’s multiple range test.

Relative electrolyte leakage (REL) is an indicator of membrane damage caused by environmental stress. To estimate the effects of heat stress-induced membrane damage in *P. ternata* leaves, REL was measured in plants after exposure to heat stress for 0.5, 2, 8, 12, 24 or 72 h. In the present experiment, REL of *P. ternata* leaf tissues, following exposure to heat stress, increased in accordance with the time of the treatments. The REL gradually increased from 6.5% to 14.3%, in heat-stress leaves, after 72 h of treatment (Figure 1A).

Malondialdehyde (MDA), a decomposition product of polyunsaturated fatty acid hydroperoxides, is a biomarker for lipid peroxidation, which is an effect of oxidative damage. The concentrations of MDA increased gradually with treatment duration, reaching a peak at the 24 h time-point and maintaining high concentration at 72 h. (Figure 1B).

Collectively, our results clearly demonstrated that the treatment regime used in this study caused marked damage to *P. ternata* leaf cells.

### 2.2. 2-DE Analysis of *P. ternata* Leaf Proteins Expressed in Response to Heat Stress

**Figure 2:**
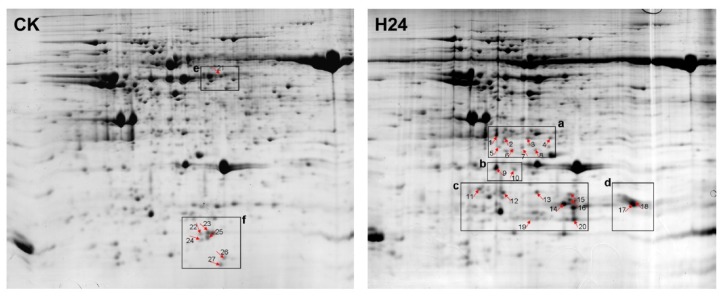
Representative 2-DE gels of *P. ternata* leaf proteins. 2-DE was performed using 1200 μg of total protein and 24 cm immobiline dry strips with linear pH gradients from pH 4–7. SDS-PAGE was performed with 12.5% gels. The gels were stained with CBB R-250. (**CK**):2-DE gel of the control sample. The down-regulated spots are numbered; (**H24**): 2-DE gel of sample treated at 38 °C for 24 h. The up-regulated spots are numbered. The framed regions **a**, **b**, **c**, **d**, **e** and **f** are enlarged in Figure 2.

**Figure 3:**
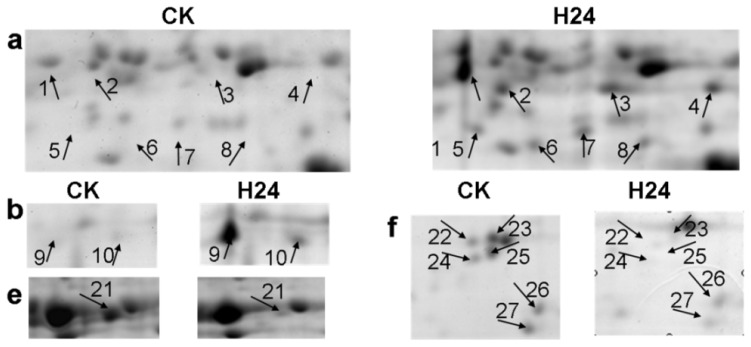

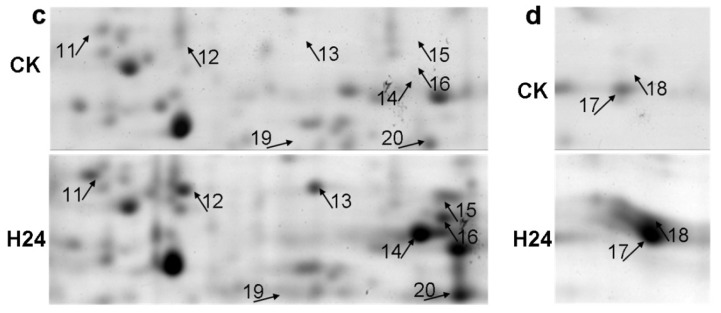
Magnified regions of differentially expressed proteins in *P. ternata* leaves.

To investigate the changes in the *P. ternata* leaf proteins in response to heat stress, samples were taken 24 h after heat treatment. We used 1200 μg of protein for the 2-DE gel analysis, which consisted of isoelectric focusing (IPG strips, 24 cm, pH 4–7) followed by SDS-PAGE (12.5%), for both the control (CK) group and the 24 h heat stress (H24) group. After electrophoresis, the gels were stained with CBB R250 and analyzed using PD Quest 7.3 software. We performed 2-DE analysis of the total proteins in *P. ternata* leaves for three biological replicates (Figure S1). More than 600 protein spots were reproducibly detected in each gel. A quantitative analysis revealed that 27 spots appeared or had changes in intensity of more than 2-fold after heat stress. Of these 27 spots, 20 were found to be up-regulated in H24 plants compared with CK plants, and seven were down-regulated (Figures 2 and 3).

### 2.3. Identification of Heat-Responsive Proteins

**Figure 4:**
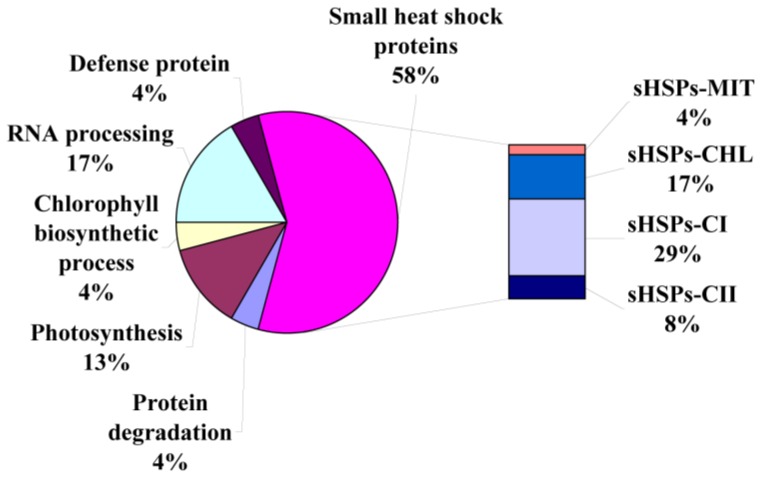
Functional groups of differentially expressed proteins identified in the control and heat-treated *P. ternata* leaves. This classification is based on their homologs and the literature. A total of six functional categories and their percentages are shown.

Proteins differentially expressed in *P. ternata* leaves in response to heat stress were identified using MALDI-TOF/TOF. As shown in Table 1, 24 proteins and protein species were successfully identified by MS/MS analysis. Based on Gene Ontology, BLAST alignments, and information from the literature, these 24 proteins and protein species were divided into six functional classes (Figure 4): small heat shock proteins (58%), RNA processing proteins (17%), photosynthesis proteins (13%), chlorophyll biosynthesis proteins (4%), protein degradation proteins (4%) and defense proteins (4%).

A total of 14 small heat shock proteins and protein species (spots 3, 4, 6, 8, 10–17, 19 and 20) were induced by heat stress. This result suggests that sHSPs may play a pivotal role in protecting the plant against cellular damage caused by heat stress. The expression levels of photosynthesis-related proteins were increased, and two chlorophyll a/b-binding protein species (spots 1 and 2) were up-regulated in H24 compared with CK plants. The Rieske Fe/S protein of the cytochrome b6/f complex (spot 18) was also expressed at a relatively high level after heat stress. Four protein species (spots 22–25) involved in RNA processing exhibited significant decreases in their abundance. One protein in the chlorophyll biosynthetic process pathway (spot 21), glutamate-1-semialdehyde 2,1-aminomutase, was down-regulated. The expression of one protein (spot 9) associated with the protein degradation pathway was significantly changed after heat stress. *P. ternata* lectin was found to have a four-fold decrease in response to heat stress.

### 2.4. Gene Expression Analysis by qPCR

To determine whether the changes in protein abundance detected by 2-DE were correlated with changes at the transcriptional level, we performed qPCR analysis. Among all the identified proteins, four proteins were selected for investigation of their mRNA expression patterns in response to heat stress. The selected proteins were sHSPs-CI, sHSPs-CII, GRP and sHSPs-MIT. Specific primers were designed based on the cloned cDNA fragments of the corresponding proteins [19]. Changes in the mRNA levels of the four genes over a time course of 0, 0.5, 2, 8, 12, 24 and 72 h at 38 °C were analyzed.

**Figure 5:**
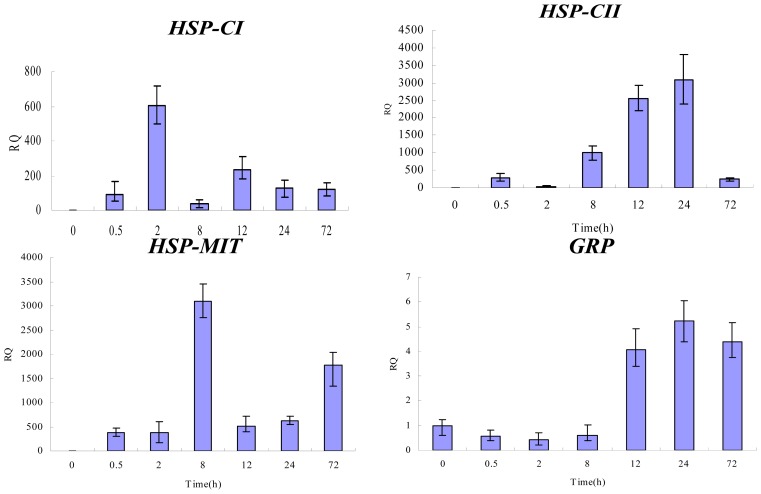
Relative gene expression levels of selected proteins in *P. ternata* leaves exposed to heat stress for 0, 0.5, 2, 8, 12, 24 and 72 h. The *GAPDH* gene was used as an internal control. The gene expression levels relative to those in the unstressed state are represented by relative quantification (RQ) values (fold changes).

As shown in Figure 5, On the whole, the expression levels of three sHSPs (*CI*, *CII*, and *MIT*) genes increased following heat stress, but different classes of sHSPs genes exhibited diverse expression profiles in response to heat stress. The mRNA level of *GRP* showed a different expression pattern. The expression of *GRP* was down-regulated at the beginning of heat stress but then increased substantially to reach a peak after 24 h of heat shock. These results provide additional support for a previously introduced concept that the mRNA level may not be consistent with the protein expression level [20].

### 2.5. Western Blot Analysis of sHSPs-CI and sHSPs-CII

**Figure 6:**
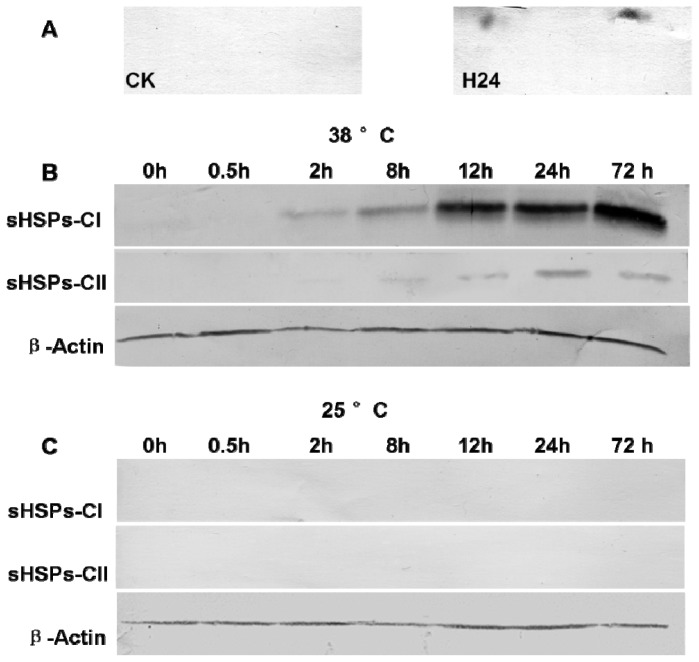
Protein level changes for sHSPs-CI and sHSPs-CII in *P. ternata* leaves exposed to heat stress. (**A**) 2-DE Western blot analysis of sHSPs-CII proteins in *P. ternata* leaves. The total proteins extracted from the unstressed (CK) or stressed samples at 24 h (H24) were separated by 2-DE. The portion of the gel corresponding to 12–18 kDa and pI 5.2–5.8 was excised; (**B**) Accumulation of sHSP-CI and sHSPs-CII proteins in the leaves of *P. ternata* induced by exposure to 38 °C; and (**C**) Accumulation of sHSP-CI and sHSP-CII proteins in the leaves of *P. ternata* maintained at 25 °C. Equal amounts (15 mg) of total protein were loaded onto sodium dodecylsulfate-polyacrylamide gel electrophoresis gels. The levels of sHSPs-CI, sHSPs-CII and actin proteins were estimated by immunoblotting, as described in the Materials and Methods Section.

In the current study, the accuracy of the 2-DE analysis was further validated by Western blot analysis. The 2-DE Western blotting was performed with the *Arabidopsis* anti-HSP17.7 CII antibody. The *P. ternata* leaf proteins from the control and stressed samples at 24 h were analyzed. The results showed that two spots could be detected by the anti-HSP17.7 CII antibody in the stressed sample, and both spots were identical to the spots identified by 2-DE (Figure 6A).

Furthermore, antibodies against *Arabidopsis thaliana* HSP17.6 CI and HSP17.7 CII were used to investigate the expression levels of sHSPs-CI and sHSPs-CII in the leaves in response to heat stress. These two classes of sHSPs (CI and CII) exhibited similar expression patterns (Figure 6B), with gradual up-regulation at the expected molecular mass of approximately 17 kD. However, The expression of sHSPs-CII reached a peak at the 24 h time-point but then declined at 72 h of heat stress. In addition, no immuno-stained bands were detected in the CK samples, and there was no change at 25 °C condition during observation series (Figure 6C).

## 3. Discussion

Heat stress leads to reduced growth and lower final yields of plants, and the negative impact of high temperatures has been reported for *P. ternata* [18,21]. Due to the ability of plants to activate a large number of stress-related genes and to synthesize a variety of functional proteins to counteract heat stress, it is important to understand the mechanism of *P. ternata*’s response to heat stress. Suppression subtractive hybridization (SSH) was used to clone transcripts that were up-regulated during heat stress in the leaves of *P. ternata*, and 44 singletons were grouped into 17 functional categories, such as responses to stress, primary metabolic processes, macromolecule metabolism and cellular processes [22]. However, most biological functions in a cell are executed by proteins and not by mRNA, and the transcription patterns are not always consistent with the protein expression levels, as shown in a previous study [20].

More needs to be known about the mechanism of action of proteins in response to heat stress. To the best of our knowledge, there have not been any proteomic analyses of *P. ternata* subjected to heat stress. Proteomics analyses of *P. ternata* have been difficult to perform due to the lack of genomic information [23]. However, cross-species identification is a powerful option for protein identification whenever a genome is poorly characterized [24,25]. In this approach, proteins are identified by comparing peptides from the proteins of interest with orthologous proteins of species that are well characterized. In this study, protein database searches were performed using all available green plant proteins in the NCBI protein databases. In the present study, the physiological data showed that the leaves of plants treated with high temperature for 24 h showed stress damage. A 2-DE-MS-based proteomic approach was used to identify proteins with different expression levels in response to heat stress. A total of 24 differentially expressed proteins and protein species were detected and functionally characterized. Many protein species were identified in more than one spot, although they were excised from the same gel. This phenomenon may result from the presence of different protein isoforms, post-translational modifications or degradation [26]. The identified proteins provide useful information on the details of the responsive mechanisms of *P. ternata* exposed to high temperatures.

### 3.1. Photosynthesis-Related Proteins

In our investigation, chlorophyll a/b-binding proteins (spots 1 and 2), the Rieske Fe/S protein of the cytochrome b6/f complex (spot 18), which are involved in photosynthesis, were up-regulated in the leaves of *P. ternata* exposed to elevated temperatures.

Chlorophyll-binding proteins are involved in harvesting light energy and transferring it to photochemical reaction centers. In the present study, two chlorophyll-binding protein species exhibited substantial increases in expression in response to heat stress. In contrast with this result, Xu and Huang [27] reported that the abundance of chlorophyll-binding proteins was down-regulated in response to drought, heat, and combined stress in both tolerant and sensitive Kentucky bluegrass cultivars, and the levels of chlorophyll-binding proteins were down-regulated to a lesser extent in the tolerant cultivar than in the sensitive cultivar. This discrepancy may be a result of the differences between the responses of *P. ternata* and Kentucky bluegrass to stress. It therefore may be more important to quantitatively analyze the chlorophyll-binding proteins in *P. ternata* leaves in response to heat stress using immunoblotting analysis in future studies.

The Rieske Fe/S protein of the cytochrome b6/f complex primarily participates in the transfer of electrons from a liposoluble quinol to a water-soluble protein, either plastocyanin or cytochrome C. The same result has been reported in several previous studies [12]. In conclusion, increasing light harvesting and the electron transfer efficiency are perhaps part of the strategy of *P. ternata* for adapting photosynthesis to high temperatures.

### 3.2. Small Heat Shock Proteins

sHSPs are highly conserved proteins of approximately 15–40 kD and are characterized by the possession of an á-crystalline domain at the *C*-terminus. sHSPs can be classified into at least six categories according to their sequence homology and subcellular localization: cytosolic class I, cytosolic class II, mitochondrial, chloroplast localized, peroxisomal and endoplasmic [28].

Several proteomic analyses have been performed to analyze the plant proteome in response to heat stress. In most cases, sHSPs belonging to the cytoplasmic sHSPs class as well as mitochondrial-targeted and chloroplast-targeted sHSPs have been observed. In the present study, 14 sHSPs were detectable only in the stressed sample. The protein sequences were analyzed with PSORT [29] to predict the proteins’ subcellular localization. To determine the categories of these identified sHSPs, we searched for homologs with BLASTP [30]. These searches combined with the sequence alignments and other data from the literature allowed these proteins to be classified.

In this study, eight spots (11–17) were identified as cytoplasmic class I family sHSPs, and spot 19 and 20 were identified as cytoplasmic class II family sHSPs. One sHSPs located in the mitochondria was present only under heat stress. Meanwhile, protein spots 3, 4, 6 and 8 were identified as homologous to chloroplast sHSPs.

The cytoplasmic sHSPs represent the most abundant sHSPs in plants [31]. Many of them act as small heat shock proteins with important functions not only in the protection of proteins against damage due to stress but also in the folding, intracellular distribution and degradation of proteins. Transgenic plants that overexpress sHSPs have improved agronomic traits with respect to basal thermotolerance [32–34]. In contrast, plants with reduced expression of sHSPs have compromised acquired thermotolerance [35].

To investigate the gene and protein expression patterns of cytoplasmic sHSPs in *P. ternata* leaves, real-time quantitative PCR and Western blot analysis were performed at different time intervals during high temperature stress at 38 °C. Using 2-DE Western blot analysis, we confirmed the identities of the sHSPs-CII identified by MS. Transcriptional profile analysis clearly showed that the *sHSPs-CI* and *sHSPs-CII* genes are highly inducible by heat stress in leaves but they exhibited diverse expression profiles in response to heat stress. The *sHSPs-CI* expression reached a peak at 2 h of heat shock but then declined at 8 h of heat stress before increasing again. The expression of *sHSPs-CII* increased gradually with treatment duration, reaching a peak at the 24 h time-point but then declined at 72 h of heat stress. In addition, the protein levels of sHSPs-CI increased with the time of heat treatment, and the sHSPs-CII exhibited the same expression patterns for their mRNA and protein levels. When the CK sample was analyzed, proteins in both classes were nearly absent. A heat shock time-course experiment was designed to analyze the influence of heat shock on the expression of *sHSPs-MIT* in *P. ternata* leaves under stressful conditions. The results are shown in Figure 5. The level of *sHSPs-MIT* transcripts increased rapidly in *P. ternata* leaves. The *sHSPs-MIT* expression reached a peak at 8 h of heat shock but then declined at 12 h of heat stress before progressively increasing again.

Chloroplast small heat shock proteins (sHSPs-CHL) are expressed in leaves in response to heat stress. *In vitro* experiments have demonstrated that sHSPs-CHL can associate with thylakoids and protect PSII during heat and other stresses, possibly by stabilizing the O_2_ evolving complex (OEC) [36]. sHSPs-MIT exhibit high similarity to sHSPs-CHL in the *C*-terminal region but differ in the *N*-terminal region of the proteins [37]. Maize sHSPs-MIT have been shown to improve mitochondrial electron transport, mainly by protecting the NADH:ubiquinone oxidoreductase activity (complex I) [38].

sHSPs are widely distributed in plants however, it will be interesting to further isolate and analyze the function of sHSPs that are unique in *P. ternata.* Heat stress strongly induces the protein expression of four different classes of sHSPs in *P. ternata* leaves. The transcripts of three sHSPs genes were increased by heat shock treatment, although with different response patterns. These results may indicate that different classes of sHSPs were regulated with different patterns or by different signals and have different assigned functions in response to heat stress. sHSPs may function together to protect the cellular machinery and are critical for the plant to tolerate heat stress.

### 3.3. Chlorophyll Biosynthetic Process Proteins

When plants are exposed to heat stress, chlorophyll biosynthesis is affected. As previously reported, after 24 h of high temperature treatment, the chlorophyll content of *P. ternata* leaves falls by 12.33% [22]. In the present study, glutamate-1-semialdehyde 2,1-aminomutase (spot 21) which is an important enzyme to catalyze the formation of 5-aminolevulinic acid (ALA) [39], was dramatically down-regulated by heat stress. In addition, ALA is the vital precursor of chlorophyll. Thus, the inhibition of chlorophyll biosynthesis is likely due to the impairment of 5-aminolevulinic acid (ALA) biosynthesis which is caused by the decreasing level of glutamate-1-semialdehyde 2,1-aminomutase.

### 3.4. Protein Degradation Proteins

One newly induced protein (spot 9) was identified as speckle-type POZ protein (SPOP). The protein sequence was subjected to Pfam analysis [40] to identify conserved domains. This protein was found to contain BTB/POZ and MATH domains. In *A. thaliana*, the BTB/POZ-MATH proteins (BPM) comprise a small family of six members. They have been described previously to use their broad complex, tram track, bric-a-brac/POX virus and zinc finger (BTB/POZ) domains to assemble with CUL3a and CUL3b and potentially serve as substrate adaptors for cullin-based E3-ligases, which are involved in the ubiquitin–proteasome pathway for protein degradation [41]. BPM has been described previously as playing a role in abiotic stress tolerance. Studies on gene expression have shown that drought, osmotic stress and salt stress can induce the expression of *BPM1*, *BPM4* and *BPM5* [42]. This result suggests that protein degradation and recycling via the ubiquitin–proteasome pathway is involved in response to heat stress in *P. ternata* leaves. The up-regulation of SPOP may promote programmed cell death and leaf senescence in response to heat stress.

### 3.5. RNA Processing Proteins

The regulation of RNA metabolism at the post-transcriptional level, including pre-mRNA splicing, capping polyadenylation, transport, turnover and translation, has been shown to play important roles in plant responses [43]. These post-transcriptional events are largely regulated by RNA-binding proteins (RBPs). The glycine-rich RNA-binding proteins (GRPs) are found ubiquitously in plants. These proteins contain a glycine-rich region at the *C*-terminus and one or more RRMs at the *N*-terminus. It has been demonstrated that the expression of GRPs can be regulated by different stresses. *ZmGRP2* is up-regulated in stressed roots of *Zea may* in an ABA-independent manner. Previous studies in *Arabidopsis* roots have indicated that *AtGRP7* is repressed in response to ABA, high salt and mannitol treatments [44]. The transcript levels of all *BnGRPs* were markedly up-regulated by cold stress. However, their expression levels were significantly down-regulated by dehydration and high salinity stress. It has been shown that *Arabidopsis grp7* mutants display impaired mRNA export from the nucleus to the cytoplasm, suggesting a potential cellular role of GRPs in mRNA export [45]. Wang *et al.* [46] reported that *LbGRP1* can enhance stress resistance by mediating physiological pathways in *Limonium bicolor* (Bunge) Kuntze.

Four spots (22–25) were identified as GRPs. All of these spots were down-regulated in the leaves of stressed *P. ternata* plants, indicative of disordered metabolism under stress conditions. The mRNA profiles of GRPs in *P. ternata* leaves after different durations of heat treatment were assessed by qPCR. The results indicate that the mRNA level is not consistent with the protein level. At the beginning of the treatment, the expression of GRPs was slightly down-regulated, but after 12 h heat stress, the expression was significantly up-regulated. This phenomenon may be caused by post-transcriptional and post-translational control mechanisms [19].

Recent studies indicate that GRPs play an important role in heat tolerance, but the exact roles that GRPs play during heat stress and the relationship between GRPs and ABA remain to be determined.

### 3.6. Defense Proteins

One protein corresponding to *P. ternata* lectin (PTL) was identified, and this protein was down-regulated by heat stress. PTL is a heterotetrameric mannose-binding lectin with three mannose-binding boxes [47]. This gene is constitutively expressed in various tissues, including tubers, leaves, stems and inflorescences [48]. Lectins in plants belong to the defense system, and most lectins play a role in the defense against different types of organisms [49,50]. The results of our present study provide a good indication that high temperature exerts a negative effect on the defense system.

## 4. Experimental Section

### 4.1. Plant Materials and Treatment

Tubers of *P. ternata* were collected from the Guizhou Academy of Agricultural Sciences (Guizhou, China) and planted in a phytotron with a relative humidity of 70%, a light intensity of 200 μmol·s^−1^·m^−2^, a 12/12 h day–night cycle and a temperature of 25 °C. Three-week-old plants were used as the source of the plant tissue for all experiments. For heat shock, the plants were moved to another phytotron maintained at 38 °C. All other conditions were identical. Control and heat-stressed plants were harvested for proteomic analysis after 24 h of heat stress. The leaves were frozen in liquid nitrogen and stored at −75 °C until extraction.

### 4.2. Physiological Parameter Measurement

Membrane damage was assessed by electrolyte leakage in leaves measured as described by Lee *et al.* [7]. 0.2 g of fresh leaf disks (1 cm diameter) were placed in tubes containing 10 mL of ultrapure water and incubated at 25 °C under light. Two hours later, the electrical conductivity of the bathing solution (*L_t_*) was measured. Next, the tubes were placed in a 25 °C growth chamber for 1 h under light, after which the electrical conductivity (*L_0_*) was measured again. Relative electrolyte leakage was calculated by the formula *L*_t_/*L_0_* × 100%. Three replicates were performed for each sample.

Lipid peroxidation was estimated by measuring the content of MDA in leaves. The MDA content was determined as described by Yang *et al.* [51]. Fresh leaves (about 0.5 g) were homogenized in 10 mL of 10% trichloroacetic acid (TCA). The homogenate was centrifuged at 12,000 *g* for 10 min. Then, 2 mL 0.5% thiobarbituric acid (TBA) in 10% TCA was added to an aliquot of 2 mL of the supernatant. The mixture was heated in boiling water for 30 min, and then quickly cooled in an ice bath. After centrifugation at 12,000 *g* for 10 min, the absorbance of the supernatant at 450, 532 and 600 nm was determined with a spectrometer (UV-2550, Shimadzu, Japan). The MDA concentration was estimated using the formula *C* (μmol^−1^) = 6.45(*A*_532_ − *A*_600_) − 0.56*A*_450_. The MDA concentration was expressed as μmol·g^−1^ freshweight. All experiments were done in triplicates (*n* = 3), and average mean values were plotted.

The physiological parameters were statistically analyzed by using Duncan’s multiple range tests. Significant differences from control values were determined at *p* < 0.05 levels.

### 4.3. Protein Extraction

Protein extraction was performed using the following method [52]. Approximately 0.2 g of fresh leaf material was ground into a powder in a mortar in liquid nitrogen and then incubated with 10 mL of TCA/acetone for at least 1 h at −20 °C. The suspension was centrifuged at 15,000 *g* for 5 min (4 °C), and the precipitate was washed twice with cold 80% acetone and centrifuged at 15,000 *g* for 5 min (4 °C). After the pellet was air-dried, 1 mL of SDS extract buffer (1.5% SDS, 0.1 M Tris-HCl, pH 6.8, 20 mM dithiothreitol (DTT), GE Healthcare, Uppsala, Sweden) was added to dissolve the precipitate, followed by centrifugation at 15,000 *g* at 4 °C for 5 min. The supernatant was mixed with an equal volume of buffered phenol (pH 8.0). The mixture was centrifuged at 15,000 *g* for 5 min, and the phenol phase was recovered. Five volumes of 0.1 M ammonium acetate/methanol solution were added and mixed with the phenol phase for 1 h at −20 °C. Then, the mixture was centrifuged at 15,000 *g* for 5 min at 4 °C. The precipitate was washed twice with cold alcohol. After being air-dried, the precipitate was dissolved in hydration solution [8 M urea, 2 M thiourea, 2% 3-[(3-Cholamidopropil) dimethylammonio]-1-propanesulfonate (CHAPS), 20 mM DTT, and 0.5% IPG buffer, GE Healthcare]. The protein concentration was measured using the Bradford method [53] and an UV-2550 spectrophotometer. The protein samples were stored at −20 °C for future use.

### 4.4. Two-Dimensional Gel Electrophoresis and Image Analysis

A dry strip of pH 4–7 (24 cm) was loaded with 1200 μg of protein for 12 h at 20 °C for isoelectric focusing using the IPGPhor III system (GE Healthcare). The isoelectric focusing (IEF) voltage was set to 500 V for 1 h, followed by 1000 V for 1 h, a linear gradient increase to 8000 V over 3 h and then 8000 V for a total of 80,000 V·h. After the first dimension was run, the IEF strips were equilibrated for 15 min with an equilibration buffer (0.1 M Tris-HCl pH 8.8, 2% SDS, 6 M urea, 30% glycerol, and 0.1 M DTT) and then incubated with the same buffer containing 2.5% iodoacetamide rather than DTT for 15 min. The second dimension of electrophoresis was performed on a 12.5% gel. The gels were stained with 0.1% (*w*/*v*) Coomassie brilliant blue R250 (CBB) for at least 3 h and destained in 10% (*v*/*v*) acetic acid until a clear background was obtained. The stained gels were scanned using an ImageScanner (GE Healthcare). The images were processed using BioRad PDQuest v7.1 (Bio-Rad, Richmond, CA USA). Each sample was analyzed by 2-DE in at least two sample replicates and three biological replicates for further analysis. Only protein spots with significant and reproducible changes of at least 2-fold, and deemed significant by Student’s *t*-test at a level of 95%, were accepted as differentially expressed. The standard error (SE) was calculated from at least three spots in replicate gels.

### 4.5. Tandem Mass Spectrometry and Database Searches

Differentially expressed protein spots were manually excised, reduced with 10 mM DTT, alkylated with 50 mM iodoacetamide and digested with 20 μL of 50 mM NH_4_HCO_3_ containing 0.01 mg/mL sequencing-grade modified trypsin (Promega, Madison, WI, USA) for 16 h at 37 °C. The supernatants were lyophilized and dissolved in 10 μL of 0.1% trifluoroacetic acid. Then, 0.5 μL of this mixture was added to a matrix consisting of 0.5 μL of 5 mg/mL 2,5-dihydroxybenzoic acid in water:acetonitrile (*v*/*v* 2:1).

The MS together with MS/MS spectra were searched against the NCBI nr (green plants) database using the softwares GPS Explorer (Applied Biosystems, Foster City, CA, USA) and MASCOT (Matrix Science, Boston, MA, USA) with the following parameter settings: trypsin cleavage, two missed cleavages allowed, peptide mass tolerance set to ±0.2 Da, fragment tolerance set to ±0.3 Da, fixed modifications of carbamidomethyl (C), partial modification of acetyl (protein *N*-term), oxidation (M), deamidated:18O(1) (NQ), dioxidation (W). Only significant hits, as defined by the MASCOT probability analysis (*p* < 0.05), were accepted.

### 4.6. RNA Isolation and Real-Time Quantitative PCR (qPCR)

Total RNA was extracted using the Plant RNeasy mini kit (Qiagen, Valencia, CA, USA). First-strand cDNA was synthesized in a 20 μL reaction volume using the RevertAid™ First Strand cDNA Synthesis Kit (Fermentas, Vilnius, Lithuania) according to the manufacturer’s instructions. cDNA was synthesized from 1 μg of RNA with an oligo(dT)18 primer. A 2 μL aliquot of the cDNA sample diluted 1:10 (*v*/*v*) was used as the template for qPCR in a 20 μL reaction volume. qPCR was performed using SYBR^®^ Premix Ex Taq™ (Takara, Dalian, China) on a Step One™ Real-Time PCR system (Applied Biosystems).

Specific primers were designed based on the cloned cDNA fragments of the corresponding proteins [19] using Primer Premier V5.0 (Premier Biosoft International, Palo Alto, CA, USA) (Table 2). Melting curve analysis was performed to verify primer specificity. All real-time PCR reactions were performed in triplicate using *PtGAPDH* as a reference. The levels of gene expression relative to those in the unstressed state were quantified as relative quantification (RQ) values, which were calculated using the 2^−ΔΔ^*^Ct^* method.

### 4.7. Western Blot Analysis

For the Western blot analysis, proteins were isolated from the same tissue samples as used for qPCR. The samples were analyzed by SDS-PAGE or 2-DE and transferred to a PVDF membrane (Millipore, Bedford, MA. USA). The membrane was incubated with anti-Arabidopsis HSP17.6 CI (Agrisera AS07254, Agrisera, Vännäs, Sweden) or anti-HSP17.7 CII (Agrisera AS07254) antibodies diluted 1:2000. The HRP-conjugated secondary antibody (Abmart, Shanghai, China) was used at a dilution of 1:10,000. The bands were developed using 3,3-diaminobenzidine tetrahydrochloride [54]. To standardize the results, â-actin (plant, Abmart, Shanghai, China) antibody was used to confirm equal loading in the Western blot analysis.

## 5. Conclusions

The *P. ternata* responded to heat stress by changing the protein expression pattern. A number of heat tolerance-related proteins were identified with various functions, including small heat shock proteins, RNA processing proteins, photosynthesis proteins, protein degradation proteins and defense proteins. Although this study was an initial proteomic investigation of the response of *P. ternata* leaves to heat stress, it is our belief that the identification of heat-responsive proteins provides not only insights into heat stress responses but also a good starting point for further investigation of the functions of these proteins using genetic and other approaches.

## Supplementary Information



## Figures and Tables

**Table 1:** MS identification of differentially expressed Proteins in *P. ternata* leaves exposed to heat stress.

Spot No.	Protein Name	Accession No.	Organism	Theor. *M*_r_ (Da)/*pI*	Exper. *M*_r_ (Da)/*pI*	Coverage (%)	Score	Subcellular localization	Matched peptides
Photosynthesis									

1	chlorophyll a/b-binding protein	EMS50798	*Triticum urartu*	31,970/5.75	31,436/5.02	6	128	chloroplast	3
2	chlorophyll a/b-binding protein	EMS50798	*Triticum urartu*	31,970/5.75	30,489/5.09	6	128	chloroplast	3
18	Rieske Fe/S protein of cytochrome b6/f complex	CAA45705	*Nicotiana tabacum*	24,511/7.59	17,911//6.11	5	68	chloroplast	1

Small heat shock proteins									

3	small heat shock protein	AEX97053	*Copaifera officinalis*	27,275/6.97	31,012/5.29	4	89	chloroplast	1
4	small heat shock protein	AEX97053	*Copaifera officinalis*	27,275/6.97	31,151/5.38	4	89	chloroplast	1
6	small heat shock protein	AEX97053	*Copaifera officinalis*	27,275/6.97	27,867/5.14	4	54	chloroplast	2
8	small heat shock protein	AEX97053	*Copaifera officinalis*	27,275/6.97	28,714/5.34	4	54	chloroplast	1
10	mitochondrial shsp	NP_001233872.1	*Solanum lycopersicum*	23,833/6.45	24,203/5.17	9	63	mitochondrial	2
11	HSP21	ABW81098	*Cleome spinosa*	17,432/5.97	18,341/4.88	11	108	cytoplasm	3
12	17.5 kDa class I hsp	AAR25848	*Carica papaya*	17,471/5.31	18,274/5.08	11	72	cytoplasm	2
13	heat shock protein 17.9	CAA63903	*Cenchrus americanus*	17,935/5.82	18,282/5.34	9	107	cytoplasm	3
14	18.2 kDa class I hsp	P27880	*Medicago sativa*	18,154/5.81	17,845/5.55	31	403	cytoplasm	4
15	heat shock protein 17.9	CAA63903	*Cenchrus americanus*	17,935/5.82	18,065/5.60	9	107	cytoplasm	3
16	18.2 kDa class I hsp	XP_002281220	*Vitis vinifera*	17,041/5.80	17,901/5.60	11	100	cytoplasm	2
17	17.7 kDa heat shock protein	AAB63311	*Helianthus annuus*	17,662/6.19	17,839/6.11	17	175	cytoplasm	3
19	small heat shock protein	AFK28040	*Pinellia ternata*	17,231/5.52	17,346/5.30	10	62	cytoplasm	1
20	small heat shock protein	AFK28040	*Pinellia ternata*	17,231/5.52	17,346/5.64	10	62	cytoplasm	1

Chlorophyll biosynthetic process									

21	Glutamate-1-semialdehyde 2,1-aminomutase	GSA_HORVU	*Hordeum vulgare*	49,690/6.39	48,096/5.84	5	62	chloroplast	2

Protein degradation									

9	putative spop	BAC66712	*Oryza sativa Japonica Group*	41,986/5.32	24,722/5.04	4	52	cytoplasm	1

RNA processing									

22	glycine-rich RNA-binding protein	XP_003631658	*Vitis vinifera*	16,376/6.32	14,987/5.62	19	62	nucleus	2
23	glycine-rich RNA-binding protein	ACJ11730	*Gossypium hirsutum*	17,075/7.82	15,011/5.67	16	204	nucleus	4
24	glycine-rich RNA-binding protein GRP1A-like	XP_003631658	*Vitis vinifera*	16,376/6.32	14,946/5.62	19	198	nucleus	4
25	glycine-rich RNA-binding protein	AAT85299	*Oryza sativa*	15,949/6.29	14,963/5.67	8	79	nucleus	2

Defense protein									

26	lectin	AAP20876	*Pinellia ternata*	29,720/6.58	11,530/5.79	11	180	extracellular	4

**Table 2:** Real-time quantitative PCR primer sequences.

Gene Name		Sequence
*sHSP-CII*	-F	5′-CGATCACACAGCACTCAACAAC-3′
	-R	5′-GCTCTCCAGTCCCAACATCC-3′

*GRP*	-F	5′-CTTCGGCTTCGTGACCTTT-3′
	-R	5′-GGTTGACGGTGATGCTCCT-3′

*sHSP-MIT*	-F	5′-GGTGGAGCAGAACACTTTGG-3′
	-R	5′-GCACCCCGTTCTTCATCTC-3′

*GAPDH*	-F	5′-TGCTGGGAATGATGTTGAATG-3′
	-R	5′-TTGGCATTGTTGAGGGTTTG-3′

*sHSP-CI*	-F	5′-GACTCGGAGACCTCTGCTTT-3′
	-R	5′-TCACTTCCTCCTTGTTGACG-3′

-F, forward primer; -R, reverse primer.
