# Supplementary material for: A Comparative Proteomic Analysis of Pinellia ternata Leaves Exposed to Heat Stress
Source: Int J Mol Sci. 2013 Oct 15;14(10):20614–34. doi: 10.3390/ijms141020614 (PMC3821634; doi:10.3390/ijms141020614)
Supplement: Supplementary file 1 [file ijms-14-20614-s001.pdf]

Supplementary Information

Figure S1. 2-DE gels of *P. ternata* leaves in control group (**CK**) and 24h heat treatment group (**H24**).

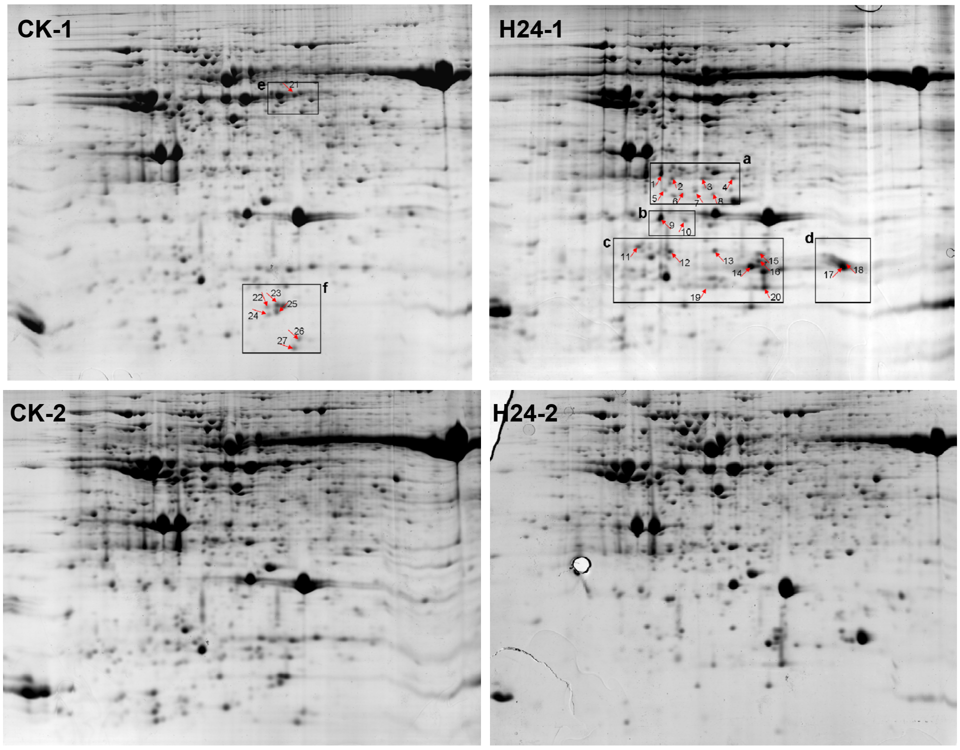

Replicate 1

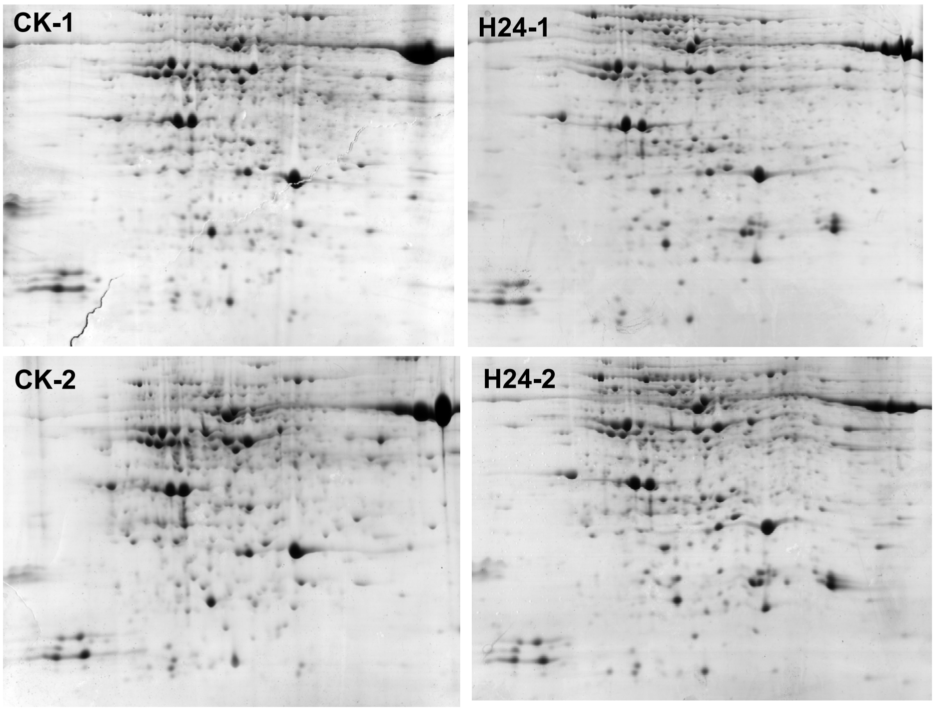

Replicate 2

Figure S1. *Cont.*

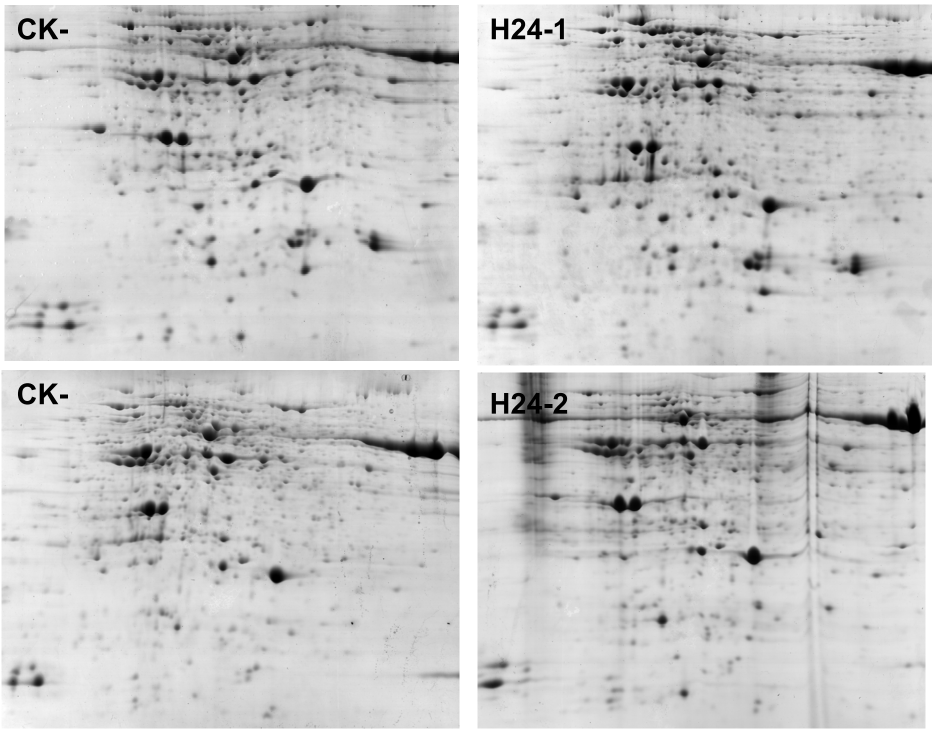

Replicate 3

© 2013 by the authors; licensee MDPI, Basel, Switzerland. This article is an open access article distributed under the terms and conditions of the Creative Commons Attribution license (http://creativecommons.org/licenses/by/3.0/).
